# Instruments to measure patient experience of health care quality in hospitals: a systematic review protocol

**DOI:** 10.1186/2046-4053-3-4

**Published:** 2014-01-04

**Authors:** Michelle Beattie, William Lauder, Iain Atherton, Douglas J Murphy

**Affiliations:** 1 School of Nursing, Midwifery and Health, University of Stirling, Highland Campus, Centre for Health Science, Old Perth Rd, Inverness IV2 3JH, UK; 2 School of Nursing, Midwifery and Health, University of Stirling, Stirling FK9 4LA, UK; 3 Quality, Safety and Informatics Research Group, University of Dundee, Mackenzie Building, Kirsty Semple Way, Dundee DD2 4BF, UK

## Abstract

**Background:**

Improving and sustaining the quality of care in hospitals is an intractable and persistent challenge. The patients’ experience of the quality of hospital care can provide insightful feedback to enable clinical teams to direct quality improvement efforts in areas where they are most needed. Yet, patient experience is often marginalised in favour of aspects of care that are easier to quantify (for example, waiting time). Attempts to measure patient experience have been hindered by a proliferation of instruments using various outcome measures with varying degrees of psychometric development and testing.

**Methods/Design:**

We will conduct a systematic review and utility critique of instruments used to measure patient experience of health care quality in hospitals. The databases Medical Literature Analysis and Retrieval System Online (MEDLINE), Cumulative Index to Nursing and Allied Health Literature (CINAHL), Psychological Information (Psych Info) and Web of Knowledge will be searched from inception until end November 2013. Search strategies will include the key words; patient, adult, hospital, secondary care, questionnaires, instruments, health care surveys, experience, satisfaction and patient opinion in various combinations. We will contact experts in the field of measuring patient experience and scrutinise all secondary references. A reviewer will apply an inclusion criteria scale to all titles and abstracts. A second reviewer will apply the inclusion criteria scale to a random 10% selection. Two reviewers will independently evaluate the methodological rigour of the testing of the instruments using the Consensus-based Standards for the Selection of Health Measurement Instruments (COSMIN) checklist. Disagreements will be resolved through consensus. Instruments will be critiqued and grouped using van der Vleuten’s utility index. We will present a narrative synthesis on the utility of all instruments and make recommendations for instrument selection in practice.

**Discussion:**

This systematic review of the utility of instruments to measure patient experience of hospital quality care will aid clinicians, managers and policy makers to select an instrument fit for purpose. Importantly, appropriate instrument selection will provide a mechanism for patients’ voices to be heard on the quality of care they receive in hospitals.

PROSPERO registration CRD42013006754.

## Background

Improving and sustaining the quality of hospital care experienced by patients continues to be a challenge worldwide [1-4]. Current quality improvement thinking advocates the use of measurement to determine whether change initiatives are indeed improving care [2,3]. Measurement, however, is difficult and no single measure can capture the multitude of facets and outcomes of modern, complex health care systems. The net result has been a proliferation of instruments to measure quality of care.

It is important to establish what constitutes quality of care from the perspective of patients, as well as having the views of clinicians and health care managers, as views differ [5]. Patients, through their unique experience, can offer insights into hospital quality that would be unseen from other perspectives, such as the way a treatment, process or interaction has made them feel and, subsequently, behave. Yet, the majority of measurement plans only include aspects of quality defined from the perspectives of clinicians and managers. Despite efforts to improve hospital care, the challenge of assuring and improving health care in hospitals remains. There is the potential that measuring and acting on issues of quality raised by patients can be a solution to this intractable problem. There is also increasing evidence that patients who have positive health care experiences have improved outcomes [6] resulting in a more efficient health care system [7]. The necessity to hear the patients’ perspective is not new. However, recent aspirations for ‘person-centred’ care and ‘mutual’ health care services [3,8] have reaffirmed the imperative for clinicians and health care managers to listen to patients’ experiences and act on them to implement improvements.

However, attempts to assess the quality of hospital care by measuring patient experience are challenging. Firstly, there is confusion over the terms ‘experience' , ‘perception’ and ‘satisfaction’; [5,7] secondly, what constitutes quality within existing instruments is not always defined from the patient’s perspective (validity); [9] thirdly, instruments need to produce consistent and reproducible results (reliability) and, essentially, instruments need to be usable in real world practice [10].

First, confusion over the terms ‘experience' , ‘perception’ and ‘satisfaction’ often result in these being used interchangeably, despite known limitations of using satisfaction as a measure of quality [11-14]. Satisfaction has been defined as the gap between a patient’s expectations and the actual care he or she received [15]. Yet, many factors influence patients’ expectations and these are not static, which threatens the validity of using satisfaction as an outcome measure. Patients do not readily express dissatisfaction with the actual care received for fear of reprisal or because of feeling empathy for those providing frontline care [16,17]. It is thought that a more accurate account of quality of care can be captured if questionnaires are designed around what patients have actually experienced, as opposed to their opinions of the experience [7,18,19]. We need to distinguish between instruments measuring patient experience and those measuring satisfaction/perceptions.

Secondly, instruments attempting to measure a patient’s experience of hospital quality care need to do just that. There needs to be sound theoretical and empirical evidence that instruments have been constructed that are representative of patients’ views of quality of care (content validity). There are multiple definitions of what constitutes quality of care and views differ between those providing and receiving health services [20-22]. There is a risk that people, with good intent, have developed instruments from supposition about important aspects of quality to patients. We need to determine the validity of existing instruments purporting to measure patient experience of hospital care.

Thirdly, instruments measuring patient experience of hospital quality care need to produce consistent and reproducible results if they are to be trusted in practice (reliability). Data arising from such an instrument may be used to direct limited resources therefore; the results need to be credible. A recent literature scan highlighted that many studies utilising instruments to measure patient experience provided limited information on their reliability and validity [5]. It is also unlikely that patient feedback instruments developed in-house would have undergone any reliability testing. There is an element of futility in employing an unreliable instrument to help deliver quality hospital care more reliably.

Importantly, instruments need to be usable in real world practice otherwise their sustainability, and therefore their purpose, will be jeopardised [10]. Instruments measuring the patients experience must be acceptable and interpretable to both patients and clinicians. The length and coherence of the instrument needs to be considered to ensure maximum returns and an adequate sample size. The skills required to score and interpret the results of the instrument are another consideration, to ensure timely feedback and use of the findings. Also important is the financial cost of instrument administration, interpretation and feedback mechanisms. These practicalities need to be balanced with other aspects of utility. For example, we know that the more items or questions an instrument contains, the more likely we are to be measuring the construct under enquiry (construct validity). Yet, instruments with multiple questions will be less easy to use in clinical practice due to the length of time it takes for patients to complete them and for staff to analyse and interpret them. There are balances and trade-offs to be made to identify an instrument fit for purpose.

The utility index developed by van der Vleuten [23] provides a useful framework to enable selection of the right instrument for the right purpose. The index consists of five components, namely; validity, reliability, educational impact, cost efficiency and acceptability. The importance of each component is largely dependent upon the purpose of the instrument. For example, an instrument measuring patient experience of hospital quality care to determine the performance rating of a hospital would likely weight more importance on reliability and validity; whereas an instrument used to provide team feedback for improvement would require an emphasis on educational impact, cost efficiency and acceptability. Where the outcome is associated with high stakes, evidence of validity and reliability are required, potentially to the detriment of other aspects of utility. To make a judgement on an instrument measuring patient experience of hospital quality, it is essential, therefore, to establish its intended purpose.

Measuring and acting on patient experience could offer a solution to the complex problem of improving the quality of hospital care. There is a necessity to balance these empirical and theoretical issues to be able to select the right instrument for the right purpose in the real world. There is a need to identify the range of instruments available to measure patient experience of health care quality, to establish the instruments intended use and assess all aspects of utility. To our knowledge there has been no previous systematic review to determine the utility of instruments to measure patient experience of health care quality in hospitals. There is, therefore, a clear gap in the existing literature, necessitating the proposed review.

### Study aim and objectives

The aim of this study is to systematically review and critique the utility of instruments available to measure patient experience of health care quality in hospitals. Study objectives are to:

1. Identify the range of instruments available to measure patient experience of hospital care.

2. Determine the intended use of the results of the instrument.

3. Examine the theoretical basis for each instrument.

4. Determine the reliability and validity of each instrument to measure patient experience of hospital care.

5. Categorise instruments according to purpose and outcome of utility critique.

6. Make recommendations on the use of existing patient experience instruments for policy, practice and research.

## Methods/Design

### Study method

A systematic review will allow relevant instruments to be identified, evaluated and summarised. This will enable efficient and accessible decision-making of instrument selection to measure patient experience of the quality of hospital care. The review will follow the Preferred Reporting Items for Systematic Reviews and Meta-Analysis (PRISMA) flow diagram and guidance set out by the Centre for Reviews and Dissemination [24,25].

### Search strategy

We are aiming to identify published instruments measuring patient experience of general hospital care. Therefore, combinations of key words (with appropriate truncation) will be devised in relation to the population (that is, adult patient), context (that is, hospital, secondary care, care setting), measure (that is, questionnaires, health care surveys, instrumentation) and outcome of interest (that is, patient experience/perspective or opinion). The following databases will be searched: Medical Literature Analysis and Retrieval System (MEDLINE), Cumulative Index to Nursing and Allied Health Literature (CINAHL), Psychological Information (Psych Info) and Web of Knowledge from their inception until July 2013. As per Centre for Review and Dissemination (CRD) Guidance a sample search strategy from MEDLINE is presented below (see Table 1). Experts in the field of measuring patient experience will also be contacted or their websites searched to identify any relevant studies. Duplicate studies will be removed using RefWorks and double checked by one researcher.

**Table 1:** Search strategy Ovid MEDLINE(R)

	**Advanced search**
1	Patient-centred care/
2	Exp *quality indicators, health care/
3	is.fs.
4	*“Process assessment (health care)”/
5	*“Health care surveys”/is (instrumentation)
6	Patient-reported.mp.
7	*“Questionnaires”/st (standards)
8	Quality of care.mp.
9	Health care surveys/ or questionnaires/
10	Patient experience.mp.
11	*“Outcome assessment (health care)”/
12	*“Inpatients”/
13	is.fs. or measure*.mp. or validation.mp.
14	Inpatients/
15	Secondary care/
16	Hospital*.mp.
17	(Acute adj (service* or care or setting*)).mp.
18	(Patient* adj3 experience*).mp.
19	(Quality* adj3 (care or healthcare)).mp.
20	1 or 10 or 18
21	14 or 15 or 16 or 17
22	5 or 13
23	20 and 21 and 22
24	2 or 8 or 19
25	23 and 24
26	(Patient* adj2 (perspective* or opinion* or experience*)).mp.
27	25 and 26

Footnote for Table 1: An asterisk (*) represents the most significant concept in Medical Subject Headings within MEDLINE. The slash (/) is used to describe more completely an aspect of a subject. A major topic asterisk before a subheading (/) indicates that that major concept and subheading are associated.

### Inclusion criteria

A reviewer will apply an inclusion criteria scale to all titles and abstracts. A second reviewer will apply the inclusion criteria scale to a random 10% selection. Disagreements will be resolved through consensus. We will ascertain the level of inter-reviewer agreement by calculating Cohen’s kappa statistic. As the result of instrument selection from the review could be used for high stakes purposes (that is, ranking in hospital ratings league tables) we would aim for a high level of agreement (k >0.8) [26] If agreement of the 10% falls below a high standard (k <0.8), a second reviewer will screen the remaining 90%. If this high threshold is not met with two reviewers, we will consider the feasibility of increasing the number of reviewers, or make the level of agreement explicit whilst acknowledging the limitations of increased error. Where decisions are unable to be made from title and abstract alone, we will retrieve the full paper. An Inclusion Selection Form has been devised to ensure standardisation of this procedure (see questions below). This form has been designed on a criteria scale basis; therefore, if the reviewer answers ‘no’ to the first question, the paper is rejected. This approach will enable progression to further inclusion questions only as necessary, thus enabling a speedy, yet thorough and transparent process. All exclusion decisions will be documented in a tabulated form. Secondary references will be scrutinised for additional instruments not identified in the literature search.

### Inclusion selection questions

1. Does the study test the psychometrics, theoretical development, or use of an instrument?

Yes  Go to question 2 No  Reject

2. Is the context of the study a hospital?

Yes  Go to question 3 No  Reject

3. Is the population adult in-patients in general surgery or medicine?

Yes  Go to question 4 No  Reject

4. Is the tool measuring the patients’ perspective, as opposed to staff or others?

Yes  Go to question 5 No  Reject

5. Is the tool in relation to hospital care as opposed to being condition specific i.e. quality of osteoporosis care?

Yes  Go to question 6 No  Reject

6. Is the tool measuring general experience as opposed to satisfaction with a specific profession, i.e. nursing?

Yes  Go to question 7 No  Reject

7. Is the tool measuring the patients’ experience, as opposed to satisfaction?

Yes  Retain paper No  Reject

Studies that meet the following inclusion criteria will be retained:

• Date: We will search retrospectively to the database inception to ensure we examine all catalogued papers available in this field.

• Language: Studies in the English language. Studies reported in a language other than English will be excluded due to translation costs.

• Study Type: Studies that examine the theoretical or conceptual background or psychometric properties of an instrument measuring patient experience of health care quality in hospitals.

• Setting: Instruments that have been tested in a hospital setting, including general surgery or medical ward/facility. Thus, instruments developed and tested in primary care, out-patient centres and other day care clinics will be excluded. Also, we will exclude areas specific to psychiatric or learning disabilities as they would be likely to need instruments developed specific to their needs. We will also eliminate instruments designed specifically for specialist areas such as intensive care, obstetrics and palliative care, as patients in highly specialised areas would be likely to have different determinants of what constitutes quality of care.

• Participants: Only adult inpatients will be included. We will, therefore, exclude instruments devised for the paediatric or neonatal population.

• Global experience of hospital care: Instruments that aim to measure patient experience of their general hospital care. Thus, condition- or procedure-specific instruments will be excluded (for example, those used to measure aspects of osteoporosis or surgical care). Whilst instruments such as Patient Reported Outcome Measures (PROMS) [27] and Patient Reported Experience Measures (PREMS) are important to determine whether patients have received optimum specialist care and treatment, they will not provide a global measure of patient hospital experience.

• Patient experience: We are keen to identify instruments that measure quality from patient experience of direct care. There are a multitude of questionnaires to measure patient satisfaction; however, we intend to exclude these due to the methodological limitations identified earlier in this paper.

• Defining quality: We will include all definitions or conceptions of quality if they have been devised from the patients’ perspective. Exploring how instruments have derived at a definition of quality will be an important critique in terms of instrument validity. Ensuring the patient is the subject of interest will remove studies that utilise practitioners' , families’ and carers' , or even managers’ definitions of health care quality.

### Data extraction

A Data Extraction Form will standardise the information recorded and aid analyses. The Data Extraction Form includes study characteristics and the five aspects of van der Vleuten’s utility index. Two researchers will independently extract the data for all included studies and agree, through consensus, the accuracy and completeness of the data. Where consensus is difficult to achieve we will use a third researcher to reach agreement (Table 2).

**Table 2:** Data extraction form

**General information**	Author
	Year
	Country of origin
	Papers
**Instrument detail**	Outcome measure
	Purpose/use instrument
	Number and type of categories
	Number of items
	Scale design
	Type of patients
	Type of environment
**Utility characteristics**	Validity
	Theoretical/conceptual framework
	Types of validity tests conducted and results
	Reliability
	Type of tests conducted and results
	Response rate
	Sample size
	Educational impact
	Ease and usefulness of interpretation
	Feedback mechanism
	Cost efficiency
	Number of raters required to detect difference
	Level of expertise required for scoring and analysis
	Acceptability
	Content validity outcomes- appropriateness of language
	Time required to compete the instrument
	Timing of administration
	Mode of administration (that is, self-completion)
	Acceptability by clinical teams and managers

#### Assessment of study quality

We will apply the Consensus-based Standards for the Selection of Health Measurement Instruments (COSMIN) checklist to evaluate the methodological rigour and results of the instruments [28-30]. The checklist has been designed by international experts in the field of health status measurement, but is equally applicable to measuring elusive concepts, such as experiences of hospital care quality. One of the main purposes of the checklist is to evaluate the methodological rigour of instruments for a systematic review [31]. The checklist is made up in modular fashion that enables specific criteria to be applied to certain tests. It is highly likely that one instrument may have several associated studies. The flexibility of various checklists ensures that the same level of scrutiny is applied to judge various studies of instruments, even if they have conducted different validity and reliability tests. See the section ‘Judging reliability and validity’ for further explanation on implementation of the COSMIN checklist.

Using the information from the Data Extraction Form and results of the application of the COSMIN checklist we will determine the relative importance of each utility item by categorising them as essential, desirable or supplementary (see detail of Utility Index Matrix below). This will enable instruments to be grouped according to purpose and comparisons made with similar instruments. This judgement will be determined by two reviewers through consensus. An independent, third person will be used to arbitrate where necessary. As this will require individual judgement we will ensure our decision-making is explicit in an accompanying narrative.

### Application of van der Vleuten’s Utility Index Matrix

Each Instrument would be judged (dependent upon extent of testing and purpose) with the following criteria and rated as essential, desirable or supplementary

Purpose

Validity

Reliability

Educational Impact

Cost Efficiency

Acceptability

### Instrument detail

We will need to know how the instrument was administered and used in order to assess the risk and type of measurement error to determine whether psychometric testing was sufficient. For example, we know that the timing of a questionnaire is likely to affect the patient’s recall of his/her hospital experience; hence this is a potential source of measurement error. Therefore, if an instrument is measuring patient experience of hospital quality care at three months post-discharge we would expect some testing to determine the stability of the instrument over time (for example, test-retest reliability).

### Examining instrument theoretical development

The theory of psychological measurement begins with identification and examination of the theoretical/conceptual development of an instrument, known as content validity. Where the theory underpinning the construction of an instrument is not presented we will search reference lists in an attempt to locate relevant/associated papers. Where evidence of theoretical or conceptual development is not evident we will report this finding. We will critique whether the development of the instrument was informed from the patients’ perspective of quality and comment on whether the process of content validity included a theoretical construction and quantification as identified by Lynn (1986) [32].

### Judging reliability and validity

**Figure 1:**
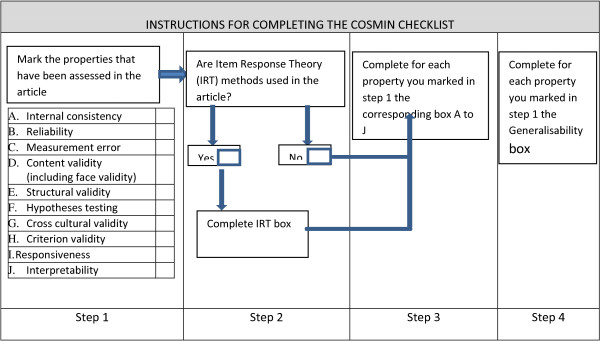
The four step procedure to complete the COSMIN checklist.

Determining what constitutes sufficient psychometric testing is complex as validity and reliability are matters of degree, as opposed to ‘all or nothing.’ However, whilst accepting that psychometric results are dependent upon the purpose, theory and number of items within an instrument, it is also important to establish the rigour of the studies conducted. We will examine the extent of the validity and reliability testing using the COSMIN checklist (see Figure 1). The checklist is applied in a four step process. Firstly, the properties that are being assessed in the study are selected, for example, internal consistency. Secondly, statistical methods used in the study are assessed to distinguish between Classical Test Theory (CTT) and Item Response Theory (IRT). For those using IRT this checklist should be completed. Thirdly, the appropriate checklist is applied depending on type of assessment determined in step one. The checklists contain relevant questions to rate the standards for methodological quality. The final step is to complete the generalizability checklist for each property identified in step one. Using the quality criteria set out by the COSMIN expert group [33] we will classify individual studies of instruments as rating positive, indeterminate or negative. The COSMIN checklist does not quantify an overall quality score as this would wrongly assume that all quality criteria have equal importance [33].

Again, the checklist will be applied by two reviewers independently before they meet to discuss and agree collectively. We will not be excluding studies on the basis of this evaluation. Rather, we will report on all the instruments we have critiqued, as the purpose of the review is to identify and assess the utility of all instruments measuring patient experience of hospital quality of care.

### Data analysis

Where applicable, we will use the general framework and specific tools outlined in the ESRC Guidance on the Conduct of Narrative Synthesis in Systematic Reviews [34]. Numerical counts will be presented to describe general information and instrument detail. We will present individual results of the COSMIN checklist application and the individual study results. We will then collectively compare and contrast instruments with similar purposes for their quality rigour and results. It would be inappropriate to conduct a meta-analysis of results of different instruments due to the variations in the way they are utilised and other heterogeneous conditions. There is currently no empirical method to pool together results of measurement properties; therefore synthesis is recommended [33]. We will categorise instruments with similar purposes and explore the individual and collective findings of application of the utility index. Given that the balance of utility is complex and specific to the function of each instrument, the analysis will be presented as a narrative synthesis. A narrative synthesis of instrument purpose, rigour and findings will enable recommendations to be made on the selection of patient experience measures for policy, practice and future research.

## Discussion

Improving and sustaining health care within hospitals continues to challenge practitioners and policy makers. Patients have unique insights into the quality of care in hospitals, but as yet are an underutilised resource in terms of measurement of quality health care. This systematic review of the utility of instruments to measure patient experience of hospital quality care will enable clinicians, managers and policy makers to select a tool fit for purpose. Ensuring this difficult, yet essential perspective of quality is included could divert resources to improve aspects of care that are important to patients. Harnessing their experience could offer the leverage needed for improvements in the quality of hospital care. We believe that this systematic review is timely and will make a valuable contribution to fill an existing research gap.

## Abbreviations

CRD: Centre for review and dissemination; PRISMA: Preferred reporting items for systematic reviews and meta-analysis.

## Competing interests

The authors declare that they have no competing interests.

## Authors’ contributions

MB conceived and designed the study, devised search strategies, drafted the inclusion selection form and drafted the manuscript. WL participated in study design, statistical advice, piloting of inclusion selection form and revision of manuscript. IA participated in study design, piloting of inclusion selection form and revision of the manuscript. DM provided direction for the study idea and design, provided statistical advice and helped revise the manuscript. All authors have read and approved the final manuscript.
